# HIV-Associated Disseminated Emmonsiosis, Johannesburg, South Africa

**DOI:** 10.3201/eid2012.140902

**Published:** 2014-12

**Authors:** Wesley G. van Hougenhouck-Tulleken, Nectarios S. Papavarnavas, Jeremy S. Nel, Lauren Y. Blackburn, Nelesh P. Govender, David C. Spencer, Christopher K. Lippincott

**Affiliations:** Helen Joseph Hospital, Johannesburg, South Africa (W.G. van Hougenhouck-Tulleken, N.S. Papavarnavas, J.S. Nel. D.C. Spencer);; University of the Witwatersrand, Johannesburg (W.G. van Hougenhouck-Tulleken, N.P. Govender);; Right to Care, Johannesburg (N.S. Papavarnavas, C.K. Lippincott, D.C. Spencer);; National Health Laboratory Service, Johannesburg (L.Y. Blackburn);; National Institute for Communicable Diseases, Johannesburg (N.P. Govender);; University of North Carolina, Chapel Hill, North Carolina, USA (C.K. Lippincott)

**Keywords:** Emmonsia sp., emmonsiosis, HIV-associated, fungal infection, South Africa, adiaspiromycosis, fungi, tuberculosis and other mycobacteria

**To the Editor:** *Emmonsia* spp., dimorphic fungi found worldwide, cause disease mainly among lower-order mammals (*1*). Although emmonsia rarely infect humans, the fungi can cause localized granulomatous pulmonary disease (adiaspiromycosis) in immunocompetent persons (*1**–**4*). Before 2013, no association was known between emmonsia and HIV, and there was no indication that emmonsia were endemic to sub-Saharan Africa.

In 2013 a novel *Emmonsia* sp. that is closely related to *E. pasteuriana* was described. The fungus caused disseminated disease in 13 HIV-infected persons in South Africa (12 in Cape Town, 1 in Bloemfontein) (*5*). Two additional cases of disseminated emmonsiosis caused by this novel species were identified in HIV-uninfected persons (1 immunocompetent, the other immunosuppressed for renal transplantation) in Cape Town (*6*). Because these cases clustered geographically, it was suggested that this novel *Emmonsia* sp. occupies a microenvironment around Cape Town (*7*). We report 3 additional cases of disseminated emmonsiosis from Johannesburg, South Africa, 403 km from Bloemfontein and 1,400 km from Cape Town. All patients were HIV-infected and reported no travel to Bloemfontein or Cape Town.

The 3 patients were admitted to Helen Joseph Hospital between August 2012 and August 2014; all patients were male and had CD4 counts of ≤5 cells/µL at admission. Patient 1 had never received antiretroviral therapy; patients 2 and 3 had defaulted antiretroviral treatment for several months before admission. All patients had disseminated skin rash, pneumonia, anemia, and substantial weight loss; chest radiographs suggested pulmonary tuberculosis. The rash appeared as disseminated hyperpigmented scaly papules and plaques (Technical Appendix Figure 1). Patients 1 and 2 also had diarrhea and exhibited delirium. 

Laboratory investigations for patient 1 showed normocytic anemia, hyponatremia, renal insufficiency, and elevated liver enzyme levels. Patients 2 and 3 had pancytopenia, hyponatremia, metabolic acidosis, and elevated liver enzyme levels. Lumbar puncture results were unremarkable for patients 1 and 3; patient 2 had normal lumbar puncture results during a previous admission (Technical Appendix Table 1). Automated laboratory identification systems initially misidentified the *Emmonsia* sp. on blood culture as *Trichosporon* spp. (patients 1 and 3) and *Histoplasma capsulatum* (patient 2). Co-infection with *Mycobacterium avium* (patients 1 and 2) and *M. tuberculosis* (patient 2) was observed (Technical Appendix Table 2). Subsequent histologic examination of skin biopsy specimens from all patients showed granulomas and yeast-like organisms.

Antifungal drug therapy consisted of fluconazole for patient 1 and conventional amphotericin B for patients 2 and 3; itraconazole was co-administered to patient 2 (Technical Appendix Table 3). Clinical and biochemical parameters improved for all patients during the first 2 weeks of hospitalization. However, hospital-acquired pneumonia developed in patient 1, who subsequently died on hospitalization day 21, and patient 2 died of an unknown cause on day 17. Permission for autopsy was not granted for either patient. At the time of this report, patient 3 was recovering well.

The fungal isolates were not identified molecularly before patients 1 and 2 died. Sequencing of the ribosomal DNA internal transcribed spacer region of isolates from all 3 patients showed 97%–99% homology with the previously described novel *Emmonsia* sp. (GenBank accession nos. KM199781–83 and KM492927) (*5*). At admission, clinical features for patients in our study were similar to those for patients with the previously reported cases of HIV-associated emmonsiosis (*5*): all patients had rash, anemia, low CD4 count, abnormal liver enzyme levels, and chest radiographs compatible with pulmonary tuberculosis.

The patients in our study received an initial misdiagnosis. Kenyon et al. (*5*) also encountered diagnostic ambiguity in invasive fungal infection cases: over an 8-year period in South Africa, 39 cases were diagnosed as histoplasmosis on the basis of histologic findings, and only 1 was confirmed by using molecular techniques. Because laboratory services in Africa are generally weak (*8*), this trend of misdiagnosis could continue. The high death rate among patients with HIV-associated emmonsiosis (31%, 5/16 patients) may partly be explained by misdiagnoses (*5*), and it also raises questions regarding optimal treatment. Amphotericin B will likely remain the optimal empiric induction therapy for suspected cases of disseminated fungal infection among HIV-infected persons in sub-Saharan Africa, given the phylogenetic proximity of *Emmonsia* spp. to *Histoplasma* spp., the antifungal minimum inhibitory concentrations of emmonsia reported thus far (*5*), and the potential for laboratory misdiagnosis of fungal infection cases. Supplementary itraconazole may be beneficial if dimorphic fungal infection, specifically emmonsia, is clinically suspected. Regardless, early confirmatory diagnoses based on culture and histopathologic results should be aggressively pursued.

Although HIV-associated emmonsiosis was suggested to be geographically isolated to the Western Cape Province, South Africa (*7*), the ecologic niche occupied by this novel *Emmonsia* sp. probably extends throughout southern Africa. Of the 13 previously reported patients (*5*), 12 lived near Cape Town (Western Cape Province) and 1 lived in Bloemfontein (Free State Province; in the center of the country) at the time of diagnosis. The 3 additional patients reported in our study resided in Johannesburg, a geographic setting distinctly separate from the other locations. A region-wide surveillance program is needed to enhance disease identification within South Africa and to determine the environmental presence of this organism beyond South Africa’s borders.

Technical Appendix Laboratory results, culture results, treatment summaries, and images for patients with disseminated HIV-associated emmonsiosis in Johannesburg, South Africa.
