# Supplementary material for: HIV-Associated Disseminated Emmonsiosis, Johannesburg, South Africa
Source: Emerg Infect Dis. 2014 Dec;20(12):2164–6. doi: 10.3201/eid2012.140902 (PMC4257822; doi:10.3201/eid2012.140902)
Supplement: Technical Appendix — Laboratory results, culture results, treatment summaries, and images for patients with disseminated HIV-associated emmonsiosis in Johannesburg, South Africa. [file 14-0902-Techapp-s1.pdf]

Article DOI: http://dx.doi.org/10.3201/eid2012.140902

HIV-Associated Disseminated Emmonsiosis, Johannesburg, South Africa

Technical Appendix

Technical Appendix Table 1. Laboratory results at admission for 3 patients with HIV-associated disseminated emmonsiosis, Johannesburg, South Africa*

Laboratory investigation	Case 1	Case 2	Case 3	Reference range
CD4 count	5 cells/μL	3 cells/μL	0 cells/μL	50–2010 cells/μL
Leukocyte count	16.91×10^9/L	$1.52 \times 1\ 0^9$/L	1.84×10^9/L	$4.00–10.00 \times 10^9$/L
Hemoglobin	8.7 g/dL	11.7 g/dL	7.8 g/dL	14.3–18.3 g/dL
Mean cell volume	91 fL	90 fL	87.6 fL	83–101 fL
Platelets	523×10^9/L	74×10^9/L	122×10^9/L	$150–400 \times 10^9$/L
Sodium	121 mmol/L	111 mmol/L	128 mmol/L	136–145 mmol/L
Potassium	3.7 mmol/L	4 mmol/L	4.8 mmol/L	3.5–5.1 mmol/L
Chloride	77 mmol/L	79 mmol/L	101 mmol/L	98–107mmol/L
Bicarbonate	30 mmol/L	16 mmol/L	16 mmol/L	23–29 mmol/L
Urea	31.4 mmol/L	5.5 mmol/L	4.8 mmol/L	2.1–7.1 mmol/L
Creatinine	590 μmol/L	55 μmol/L	64 μmol/L	64–104 μmol/L
Total bilirubin	5 μmol/L	12 μmol/L	7 μmol/L	5–21 μmol/L
Conjugated bilirubin	3 μmol/L	9 μmol/L	5 μmol/L	0–3 μmol/L
Total protein	51 g/L	56 g/L	46 g/L	60–78 g/L
Albumin	11 g/L	22 g/L	13 g/L	35–52 g/L
Alkaline phosphatase	400 U/L	301 U/L	131 U/L	40–120 U/L
γ-glutamyl transpeptidase	396 U/L	228 U/L	92 U/L	0–60 U/L
Alanine transaminase	37 U/L	53 U/L	40 U/L	10–40 U/L
Aspartate transaminase	266 U/L	94 U/L	145 U/L	15–40 U/L
Hepatitis A IgM antibody	Neg	Neg	ND	–
Hepatitis B surface antigen	Neg	Neg	ND	–
Hepatitis B core IgM antibody	Neg	Neg	ND	–
Hepatitis C antibody	Neg	Neg	ND	–
Cryptococcal serum antigen	Neg	Neg	Neg	–
CSF polymorphonuclear cells	0	0†	0	0
CSF lymphocytes	0	0†	0	0
CSF erythrocytes	0	18†	30	0

*CD4, CD4+ T-cell; CSF, cerebrospinal fluid; ND, not done; Neg, negative; NG, no growth.
†Lumbar puncture results reported are from a hospitalization in June 2013; meningeal disease was not suspected during the August 2013 hospitalization when the patient was diagnosed with emmonsiosis.

Technical Appendix Table 2. Culture data for 3 patients with HIV-associated disseminated emmonsiosis, Johannesburg, South Africa*

Case	Source	Initial Identification	Final Identification	Time to Positivity
1	Blood	NTM	NTM	14 d
	Blood	*Trichosporon* spp.	*Emmonsia* spp.†	157.7 h
	Blood	*Trichosporon* spp.	*Emmonsia* spp. †	124.9 h
2	Blood	MTB and MAC	MTB and MAC	36 d
	BMA	*Histoplasma capsulatum*	*Emmonsia* spp.†	45.0 h
3	Blood	Trichosporon spp.	*Emmonsia* spp.†	168.0 h

*BMA, bone marrow aspirate; MAC, *Mycobacterium avium* complex; MTB, *Mycobacterium tuberculosis*; NTM, unspeciated nontuberculous mycobacteria.
†Final identification of *Emmonsia* spp. was accomplished by sequencing of the internal transcribed spacer of the ribosomal DNA from the fungal isolate in each case.

Technical Appendix Table 3: Summary of inpatient antimicrobial drug treatment administered

Case	Antifungal agent	Antimycobacterial agent	Antibacterial agent
1	Fluconazole†	Rifampin/isoniazid/pyrazinamide/ethambutol combination*	Amoxicillin/clavulanic acid†
2	Amphotericin B† and itraconazole*	Rifampin/isoniazid/pyrazinamide/ethambutol combination* and azithromycin*	Amoxicillin/clavulanic acid†
3	Amphotericin B†		Amoxicillin/clavulanic acid,† azithromycin,† and trimethoprim/sulfamethoxazole†

*Orally administered.
†Intravenously administered.

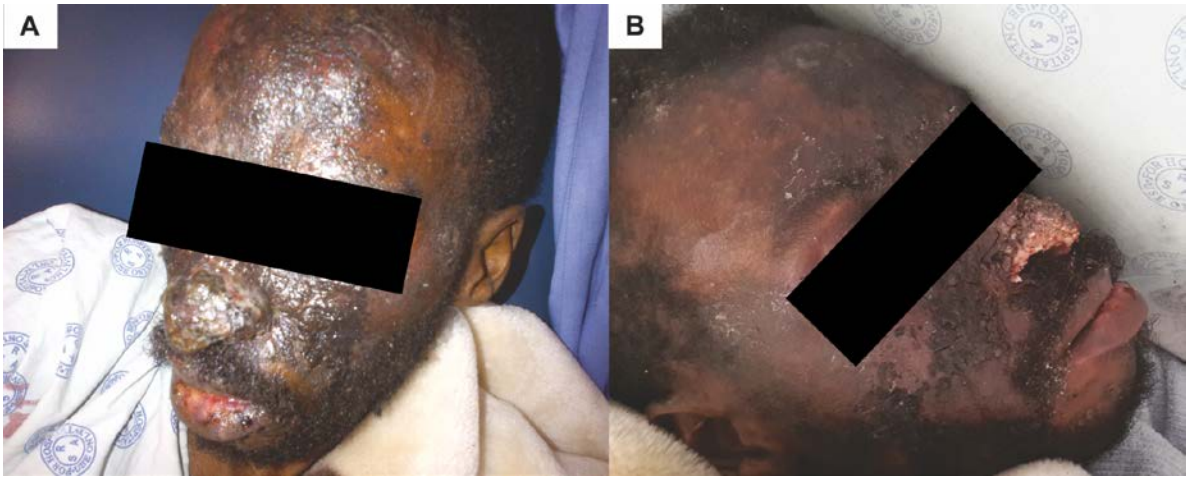

Technical Appendix Figure 1. A, Disseminated hyperpigmented scaly papules and plaques of the face with relative sparing of the eyelids is shown before treatment. B, Clinical response of the rash is shown after 10 days of treatment.

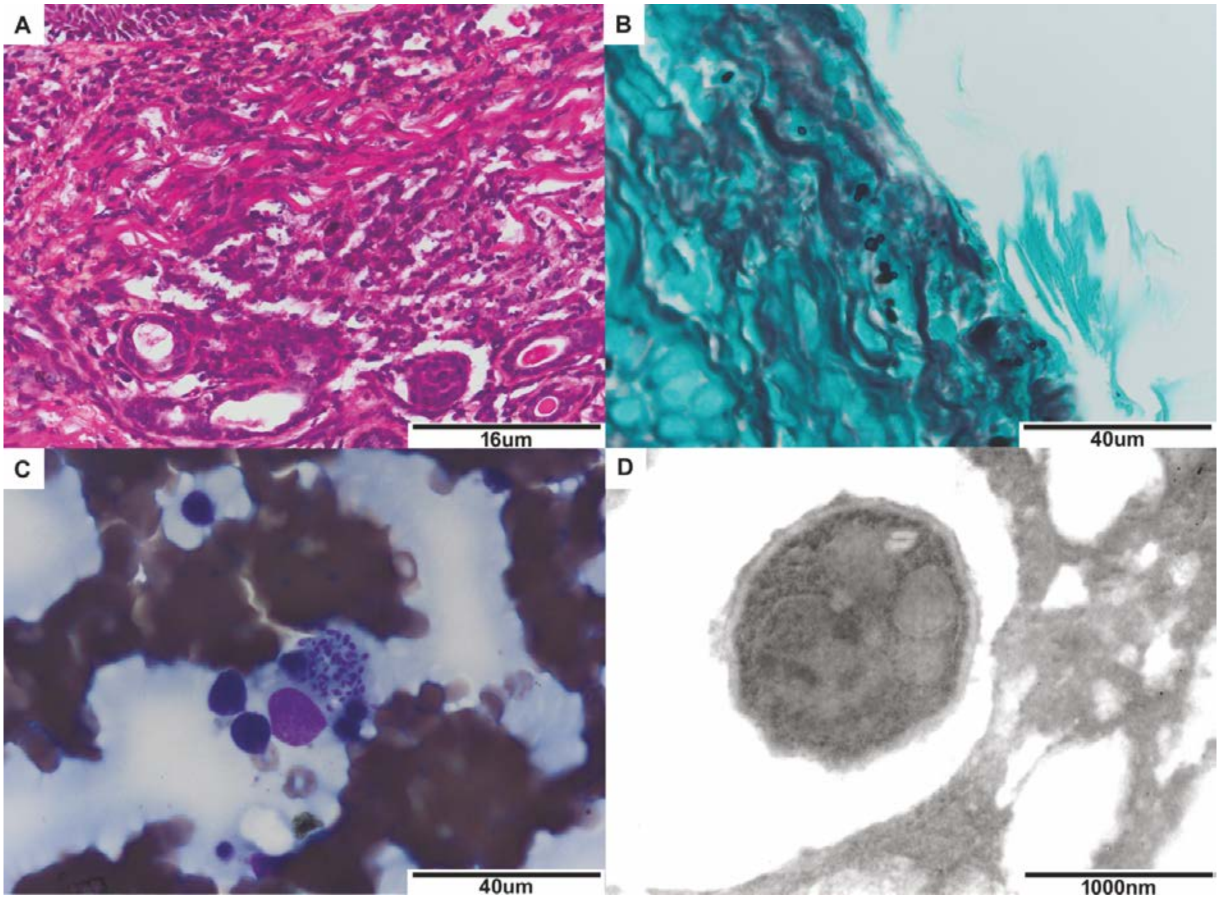

Technical Appendix Figure 2. A, Hematoxylin and eosin stain (original magnification × 40) of the skin biopsy demonstrates patchy inflammatory foci within the dermis (superficial and deep), that are predominantly suppurative, but also an occasional poorly formed granuloma. Bar represents 16 μm. B, Grocott stain (original magnification × 100) showing narrow-based budding yeasts within the dermis. Bar represents 40 μm. C, Bone marrow aspirate (100×) demonstrating a macrophage with multiple engulfed intracellular yeasts. Bar represents 40 μm. D, Transmission electron micrograph (original magnification × 40,000) of the dermis showing a free -lying organism in the yeast-phase. Bar represents 1,000 nm.

.
